# Responsibility and Laboratory Animal Research Governance

**DOI:** 10.1177/0162243917727866

**Published:** 2017-09-01

**Authors:** Carmen McLeod, Sarah Hartley

**Affiliations:** 1 University of Nottingham, Nottingham, UK; 2 University of Oxford, Oxford, UK; 3 University of Exeter, Exeter, UK

**Keywords:** engagement, intervention, politics, power, governance, expertise, other

## Abstract

The use of animals in experiments and research remains highly contentious. Laboratory animal research governance provides guidance and regulatory frameworks to oversee the use and welfare of laboratory animals and relies heavily on the replacement, reduction, and refinement (3Rs) principles to demonstrate responsibility. However, the application of the 3Rs is criticized for being too narrow in focus and closing down societal concerns and political questions about the purpose of animal laboratory research. These critiques challenge the legitimacy of responsibility in laboratory animal research governance and call for new approaches. With the advent of the "Responsible Research and Innovation" (RRI) agenda, we investigate whether the notion of responsibility in the controversial area of animal research governance could be enhanced by examining the 3Rs through RRI. Our analysis reveals RRI has the potential to helpfully augment the 3Rs in three key ways: recognizing the need to include a broader range of experts and publics in animal research governance; emphasizing the importance for animal research scientists of taking societal, and not just role, responsibilities into account; and acknowledging the political questions animal research raises.

Laboratory animals are used for researching the efficacy and safety of new medicinal products, to test biological and chemical substances, and to develop knowledge about human and animal biological processes. Laboratory animal research governance provides guidance, regulatory frameworks, and licenses to oversee the use and welfare of laboratory animals. However, the use of animals in laboratories remains a highly contentious issue and over the past four decades, there has been an increase in public skepticism and mistrust about justifications for animal experimentation to advance scientific goals (Michael and Birke 1994; Ormandy and Schuppli 2014; von Roten 2012).

In 1959, Russell and Burch first introduced the three principles of replacement, reduction, and refinement known as the 3Rs (see Kirk 2018). In the laboratory animal context, “replacement” means that conscious living higher animals must be substituted with alternative methods wherever possible, “reduction” means the number of animals used must be reduced to the minimum necessary to attain valid scientific results, and “refinement” requires the least severe procedure must be used in any experiment and animal welfare should be paramount. These principles have gradually become the foundation of animal research policy and practice in the United Kingdom (UK), the European Union (EU), and the United States and are increasingly incorporated into other governance frameworks internationally (Canadian Council on Animal Care 2015; Home Office 2013). For example, when the EU Directive *On the Protection of Animals used for Scientific Purposes* was updated in 2010, one of the key aims was to embed the 3Rs in EU legislation (European Commission 2016).

Within animal research, responsibility is linked to reassurances about how animals are used and cared for during the research process (Matthiessen, Lucaroni, and Sachez 2003) and the 3Rs are a key tool for demonstrating this responsibility. Table 1 shows how industry, research institutions, professional scientific organizations, funders, and regulators draw on the 3Rs to demonstrate responsibility. Indeed, Banks (1995) argues that responsibility should be a fourth “R” added to the 3Rs framework. However, various critics of animal research are concerned that the 3Rs are not being fully implemented. Antivivisection organizations dispute there is any usefulness in applying the 3Rs because the principles of reduction and refinement implicitly support the continued use of animals in laboratory research (House of Lords 2002; Rusche 2003). Some critics even describe the 3Rs as a smoke screen that deflects attention away from debate about the scientific validity of using animals for research purposes toward discussions about animal welfare (e.g., see Safer Medicines 2015). These critiques of the 3Rs challenge the legitimacy of the current interpretation and practice of laboratory animal research governance and call for new approaches to how responsibility is conceptualized.

**Table 1.** Evidence of the Demonstration of Responsibility through the 3Rs.

**Industry**
“It is our responsibility to use the most appropriate methodology and to aggressively seek scientifically valid 3-R approaches to animal research.” (Merck 2015) “At Lilly, we know we have both an ethical and a scientific responsibility toward animals used in research. That’s why we have adopted ‘3Rs’ when it comes to our principles of animal care and use.” (Lilly 2015) “Our commitment to the 3Rs and high standards of animal welfare begins in the Code of Conduct, and is reflected in our global Bioethics Policy.” (AstraZeneca 2015)
**Animal research institutions/professional science bodies**
“The 3Rs principles…are endorsed and incorporated by all responsible scientists.” (European Animal Research Association 2015) [The University] “…is committed to pursue a policy of reduction, replacement, and refinement (3Rs) in all animal based research and to promote knowledge of the moral and legal responsibilities and a culture of care in all aspects of research.” (University of Oxford 2015) “It is the responsibility of everyone who uses animals to ensure that they are only used when absolutely necessary and that when they are used they are treated with care and respect. If an animal is used for research, testing or teaching the work must be conducted in line with the Three Rs.” (Australian and New Zealand Council for the Care of Animals in Research and Teaching 2017)
**Regulators/funders**
“Researchers are expected to give appropriate consideration to the 3Rs in any research involving animals that has the potential to cause the animals harm and to explain in their research proposals…how the 3Rs have been taken into account.” (National Centre for the Replacement, Refinement and Reduction of Animals in Research 2014) “You must put in place systems which ensure that activities at your establishment follow the principles of the 3Rs—replacement, reduction and refinement.” (Home Office 2014, 23) “The principles of Replacement, Reduction and Refinement must be considered systematically at all times when animals are used for scientific purposes in the EU.” (European Commission 2016)

“Responsible research and innovation” (RRI) is a recent and broader approach to responsibly guide contentious scientific research. RRI builds on previous science governance frameworks with the aim of allowing for a more inclusive and adaptive approach that will ensure research outcomes are both desirable and acceptable for society (Stahl 2013). To date, no one has applied RRI to laboratory animal research. To address this gap, we investigate the potential value of RRI to enhance responsibility in the controversial area of animal research governance by examining the 3Rs through RRI. To do so, we draw on primary research conducted on the Leverhulme Trust program “Making Science Public: Challenges and Opportunities.” To further understand discourses relating to the 3Rs and constructions of responsibility that had tangentially emerged from the primary project work, we undertook a scoping study (Arksey and O’Malley 2005). Through scoping, the aim is to synthesize and analyze a broad range of academic and nonacademic materials in order to make a subject area more coherent and intelligible (Davis, Drey, and Gould 2009). Data collection for the scoping study began with four semistructured expert interviews carried out in late 2014 with UK policy makers. Three interviews were carried out face-to-face with individuals who hold senior policy posts within organizations that either fund animal research or alternatives to animal use, and one interview was carried out by phone with a senior university administrator with expertise on RRI policy. These interviews were exploratory, with the aim of identifying issues or themes which could begin to shape our analysis. A documentary analysis exercise was also undertaken, which included policy documents and other gray literature, media reports, and webpages (organizations, institutions, and industry). The majority of these data were collected electronically through search engines Google and Google Scholar and through databases such as Web of Science, Lexis Nexis, and ProQuest. In order to identify relevant texts, we applied various combinations of search terms relating to responsibility and laboratory animal research/experimentation/testing and three Rs/3Rs. The scoping materials, and insights from the aforementioned program of research, inform the conceptual and policy reflections presented here. Through our analysis, we argue that RRI has the potential to enrich the 3Rs by emphasizing inclusivity of both a broader range of experts and publics, the importance of scientists’ societal responsibilities, and the broader political dimensions of animal research.

## Responsibility, Scientists, Animals, and Society

Responsibility for the impacts of science has traditionally fallen within the professional remit of scientists, even when that science has been controversial and linked to broader societal issues (Stilgoe, Owen, and Macnaghten 2013; Pellizoni 2004). However, this narrow view of responsibility has been challenged, particularly in recent years. Douglas (2003) argues that scientists are subject to two forms of responsibility: role and general responsibilities. Role responsibility refers to scientists’ professional duties to develop scientific knowledge. General responsibility is broader, referring to scientists’ duty to consider the impact of their research outside of knowledge production, particularly in terms of societal consequences. In the UK, the role responsibilities of animal researchers can be traced back to the 1876 Cruelty to Animals Act and are embedded in policy documents (O’Donoghue 1980). For example, UK funding bodies and the National Centre for the Replacement, Refinement and Reduction of Animals in Research (2014) produced a set of guidelines entitled *Responsibility in the Use of Animals in Bioscience Research*, which set out role responsibilities for animal researchers, ethics committees, and peer reviewers to ensure implementation of the 3Rs. There is no mention of the kind of responsibilities Douglas refers to as general responsibilities. However, Douglas (2003) insists that scientists are obligated to consider the wider circumstances of their research due to their expertise and specialist knowledge. She cautions that if general responsibilities are not taken into account by scientists, they will relinquish certain aspects of their scientific freedom because other actors will determine the appropriate direction and application of research.

Like Douglas (2003), the literature on animal research governance also frames responsibility more broadly than the role responsibilities of scientists and asks us to think about humans’ responsibilities to animals. For example, Rowan and Goldberg (1995) argue that the pursuit of knowledge (role responsibilities) must incorporate an awareness of responsibilities to humanity, nonhumans, and the wider environment as a whole (general responsibilities). Similarly, Uvarov (1984) argues that as the beneficiary of laboratory animal research, society must share responsibility with scientists for animal experiments, particularly when the research is associated with pain. Haraway takes this argument further, making the case for a more embodied shared suffering with animal subjects in order to accomplish what she terms “response-ability” (Haraway 1997, 71-83). Greenhough and Roe’s (2010) review of Haraway’s thesis discusses how her work corresponds with other scholars who emphasize a shift away from the notion of individual accountability (role responsibilities) toward thinking about a much broader collective responsibility for issues relating to animals (general responsibilities; also see Greenhough and Roe 2018). Importantly, Haraway’s thesis stresses that decisions relating to animal use must be transparent (in the sense that animal suffering should be openly acknowledged), and only after this acknowledgment can collective societal responsibility be achieved for the harms and benefits of animal research.

The science and technology studies and politics literatures have also witnessed a reframing of responsibility, developing a broader and more inclusive concept capable of addressing value-based and political questions about research. For example, Owen, Macnaghten, and Stilgoe (2012) introduced RRI as a means of reframing responsibility within innovation as a collective and uncertain activity, where attention is focused on values such as care and responsiveness, rather than rules-based regulations and guidelines. RRI acknowledges the political nature of controversial science and is focused on the *purpose* of science, not just the risks. Identifying and negotiating the purpose of research is an inherently political question. They argue RRI recognizes this political dimension and may create space to discuss these political questions about the purpose and direction of research. As such, it requires a broad range of publics and/or experts to shape the direction of scientific research toward social benefits. The involvement of multiple actors enables a shared responsibility for alignments to be made between the social and the technical in shaping the direction and pace of research (see also Stilgoe, Owen, and Macnaghten 2013).

A benefit of RRI is that it offers a practical framework for action and a means to consider issues such as power, democracy, and equity. These issues are not in themselves scientific but are inherent to innovations in science and technology (Owen et al. 2013). However, it will be difficult to expand the responsibilities of actors involved in animal research and to include a broader range of voices. Franco and Olsson (2014) argue that even though laboratory animal research is strictly regulated, implementation of the 3Rs is determined by the way in which individual animal researchers’ acknowledge their responsibilities. Likewise, an examination of RRI in a UK university showed that for RRI to be successful in practice, scientific researchers must acknowledge their societal responsibilities (Hartley, Pearce, and Taylor 2017). However, the value of science for society and the economy often results in role responsibilities trumping general responsibilities (Douglas 2003). In practice, this dominance of role responsibilities may act as a way of “closing down” political and value questions in animal research governance (Stirling 2008).

## The 3Rs and RRI

In this analysis, we adopt Owen, Stilgoe, and Macnaghten’s RRI framework, which has been developed and applied in a UK academic context and widely adopted elsewhere, including by the UK’s Engineering and Physical Sciences Research Council (EPSRC; see also Owen et al. 2013; Stilgoe, Owen, and Macnaghten 2013). This RRI framework emphasizes the importance of reflexivity and inclusion throughout the life cycle of an innovation process by continuous commitment to four (interrelated) dimensions: (1) anticipation, (2) reflection, (3) inclusion, and (4) responsiveness. We will examine the 3Rs through each of the four RRI dimensions, analyzing where these two frameworks are aligned and where they are not.

*Anticipation* improves foresight of broad risk issues by encouraging researchers to think deeply and systematically about potential impacts of their research, taking into account not only opportunities but also being alert to social and ethical implications (Owen et al. 2013). In laboratory animal research, the harm–benefit analysis weighs up anticipated benefits of the research against potential harms to the animals. As an anticipatory exercise, the harm–benefit analysis has been criticized for too much focus on the promissory benefits to health and biomedicine, and not enough consideration of potential harms, as well as a lack of transparency around the ethical review process (Varga 2013). This same criticism has been levied at scientific research more broadly (Jasanoff 2003; Wynne 2011).

There is space within animal research governance for laboratory animal researchers to anticipate potential impacts of their research, specifically in relation to the 3Rs. For example, animal research is regulated under the Animals (Scientific Procedures) Act (ASPA) in the UK and each study must be covered by a project license. This licensing process is overseen by the UK Government Home Office. The project license application form includes a section requiring a description of how the researcher will comply with the 3Rs and requires justification for the use of protocols categorized as “severe.” In addition, there is now a requirement for a retrospective assessment of the actual severity of procedures experienced by animals during the course of the research (for full details of the severity classification procedures, see Home Office 2014). While this example does suggest there is at least some implementation of the aims of an anticipatory dimension, researchers are not asked to anticipate the social and ethical implications of their work beyond the 3Rs. This type of “anticipation” closes down, rather than opens up, consideration of the potential impacts. Animal laboratory researchers are only asked about a narrow range of impacts on animals and scientific outcomes and not more broadly about their general responsibilities: the *purpose* of the research remains unquestioned.

*Reflection*, or *reflexivity*, directly links responsibility within innovation practice to the obligation for researchers to reflect on the values that underlie their own work and broader governance systems, particularly critically examining the ethical, political, social, and economic assumptions that often motivate innovation processes (Stilgoe, Owen, and Macnaghten 2013). A consequence of reflexivity is greater openness within science and innovation about the uncertainties that are part of these processes (Owen et al. 2013). In animal research governance, it is important for animal researchers to be able to reflect on the moral and ethical values that are inherent to animal experimentation (Gluck and Kubacki 1991). While the majority of animal researchers are considered to be highly principled (Curzer et al. 2016), little space is allowed for reflection on personal values, or how the purpose of animal research fits within the wider sociopolitical and economic landscape particularly during the development of research protocols. Some professional organizations do encourage reflection, however. Guidance provided by the British Psychological Society (2012, 15), for example, urges psychologists who use animals to ensure they are fully informed about the debate on the “desirability of animal research.”

The current UK and EU animal research regulatory systems, like many other countries, incorporate ethics committees. In the UK, they are called Animal Welfare and Ethical Review Bodies (AWERBs). These committees provide the main space for reflection. However, researchers are not normally encouraged to reflect beyond issues of animal suffering and weighing up harms and benefits of their research. These committees could be expanded to allow an opportunity for reflection by opening up a space for animal researchers to critically evaluate the values and subjective assumptions that contribute to their decision-making and the governance of animal use more broadly. It would be productive for future research to explore how greater reflexivity could be supported and to investigate how the scientific, emotional, and ethical processes of coproduction (see Pickersgill 2012) within animal laboratory research are shaping knowledge outcomes.

*Inclusion* allows for inclusive deliberative opportunities for citizens, stakeholders, scientists, policy makers, and so on, and bringing about more shared decision-making for science and innovation governance (Stilgoe, Owen, and Macnaghten 2013). Inclusion calls for diversity and input from both publics and a broader range of experts—particularly in relation to research with the potential to impact on society (Hartley, Pearce, and Taylor 2017). The importance of including a broad range of actors has been explored in relation to controversial, emerging technologies such as nanotechnology (e.g., Guston 2013) and synthetic biology (e.g., Frow and Calvert 2013). Currently, animal research governance is expert-driven, with insufficient mechanisms and opportunities for listening to the views of other actors (Ormandy and Schuppli 2014). Scientific experts have significant influence on the development of legislative instruments, such as the UK ASPA (Lyons 2011). Broader public interests are often assumed to be represented by animal welfare organizations, such as the Royal Society for the Prevention of Cruelty to Animals (RSPCA), who have access to decision makers during the development of animal research governance frameworks (e.g., RSPCA 2011).

In the UK, public representation at the level of decision-making in relation to the approval of animal research projects is limited to lay membership of the abovementioned AWERBs. These bodies consider project license applications, including ethical issues associated with the use of animals. They are made up of scientists, animal care staff, a veterinary surgeon, and normally one independent external lay member (although the inclusion of a lay member is not mandated). The Science Media Centre, an independent press office that provides science news to the public, argues that the function of AWERBS and the ethical review process allows responsibility to be shared beyond academic and scientific communities (Science Media Centre 2013). However, relying on this approach to inclusion is wholly inadequate compared to the inclusion described by RRI. Some animal welfare organizations have called for greater public scrutiny of project license applications before they are approved (e.g., National Anti-Vivisection Society 2015), but these calls have been unheeded on the basis that the public is not qualified to scrutinize animal research proposals. Recently, there has been a push for greater transparency in animal research, which has been resisted in the past due to fears of animal rights activism. However, the relationship between transparency and inclusivity in science governance is not necessarily interchangeable. For example, while UK universities have responded to the recent Concordat on Openness on Animal Research by providing more detailed information about animal research (Petty-Saphon 2015), there is debate as to whether greater transparency does actually enable the inclusion of a broader range of actors in shaping animal research governance (Mcleod and Hobson-West 2016). Such an opening up of animal research may simply protect the autonomy and academic freedom of scientists, while continuing to close down public access to the important political questions about the purpose of research.

*Responsiveness* emphasizes the need for flexibility within research and innovation processes and the capacity to act and alter the direction of research in response to changes in social and political norms and expectations (Stilgoe, Owen, and Macnaghten 2013). Responsiveness often incorporates the three previous dimensions by ensuring that the direction and speed of innovation are determined through a governance process that includes effective and inclusive opportunities for reflection and anticipation (Owen et al. 2013). Animal research commentators also utilize the idea of responsiveness, particularly in relation to its importance for public confidence in ethical decision-making (Smith and Boyd 2007). Animal laboratory research is bound up with political issues concerning multiple competing societal viewpoints about animals and their moral status and disputes about which types of humane exploitation of animals are acceptable. This means animal researchers must legitimize their work by engaging in some form of moral argument that reflects these societal views.

The fundamental goals of the 3Rs—to incorporate social concerns into the design of animal research—can be seen as a good example of responsiveness (see Michael and Birke 1994). There are also some specific examples where changes in the moral landscape have led to political changes in the instrumental use of animals, such as the case of monkey experiments in Denmark, where the moral status of the animals changed (see Koch and Svendsen 2015). The case of UK and EU public rejection of cosmetic testing on animals is another important example of this political responsiveness, which was mainly driven by campaign organizations (e.g., European Coalition to End Animal Experiments n.d.). However, such changes are not easy or fast as animal research continues to be a contradictory, complex, and divisive topic (Ascione and Shapiro 2009). Moreover, the 3Rs are embedded within existing governance frameworks that facilitate and require research design to explicitly consider animal welfare issues and justification of the harms compared to benefits. However, these frameworks can be an obstacle to change, as they are closely aligned to established research and development processes, where economic objectives may conflict with RRI’s broader remit (de Saille 2015). While the original goal of the 3Rs was to encourage scientists to respond to and more directly include societal concerns in decision-making relating to animal research, the operation of the 3Rs––within the current regulatory system––opens up science and welfare concerns to be considered but closes down broader societal considerations.

## General Responsibility, Inclusivity, and the Political Nature of Animal Research

An examination of a 3Rs approach to responsibility in animal research governance through the lens of RRI highlights RRI’s potential both to challenge and to enhance responsibility. In addition, the case we have presented here highlights RRI’s anthropocentric concept of responsibility and care and we argue calls for greater consideration of nonhuman animals. We will explore these three points in more detail.

First, RRI seems to demand a shift from the current dominant focus in animal research governance on the role responsibilities of scientists to consideration of the societal impacts of laboratory animal research or what Douglas (2003) calls, *general responsibilities*. In thinking about these broader responsibilities, RRI usefully highlights the political nature of animal research and offers a structured way to address political issues. The 3Rs rely on laboratory animal researchers’ role responsibilities, whereas RRI requires these researchers and a broader range of actors involved in animal research governance to think about societal responsibilities. The 3Rs have been described as the metric of progress for demonstrating that the well-being of animals is taken seriously within laboratory research (Carbone 2012). However, while the scientific merits of the 3Rs are increasingly being highlighted, there is little emphasis on the societal dimensions. Instead, scientists are expected to defer questions relating to societal responsibilities to an intangible and nebulous society (Kerr, Cunningham-Burley, and Amos 1997) or the (normally) sole lay member on an ethics committee or AWERB. In other words, society and the lay public are generally held responsible for the values-based decisions made in the laboratory (Hobson-West 2012). The challenge, therefore, is to *join up* the responsibilities between broader society, laboratory animal researchers, and the governance structures.

The 3Rs framework has become a vital symbol of good science and welfare practices that allows considerable room for scientists to consider their role responsibilities. However, general responsibilities, which encompass broader political values, are not so easily incorporated. Although the application of the 3Rs opens up a process for ensuring that appropriate scientific and welfare decisions are being made within the laboratory, opportunities for deliberation about the wider sociopolitical framing and decision-making about animal use in response to human health and medical issues are closed down (Stirling 2008). This is especially pertinent in relation to questions about *who* is able to take responsibility for decision-making on the governance of animal research.

Second, the analysis highlights the importance of *inclusivity* to responsibility, particularly the inclusion of publics and experts in decision-making about animal research. This inclusivity could help broaden the 3Rs’ narrow focus on science and welfare to include discussion of the purpose of animal research. The controversial nature of animal research challenges what counts as responsible and legitimate science (Rupke 1987; Tester 1991) both in a general sense and when operationalized through the 3Rs. In the UK, animal rights “extremism,” coupled with exposés of unethical behaviors within some institutions, has created what the head of Animals in Science Regulation Unit terms a “vicious circle of mistrust” between scientists and wider society (MacArthur Clark 2015). This history continues to impact on the decision-making of scientists and policy makers (see McLeod 2018). However, it also highlights the need for opportunities for inclusive discussions about animal research that are not limited to scientific questions. Guston (2013) argues that the inclusion of previously overlooked voices within the governance of technology will not necessarily lead to consensus but can lead to more humane and legitimate ends. In the context of animal research, Olsson et al. (2012) argue that disagreements over the purpose of animal research and the values underlying the 3Rs reinforces the need for a deliberative process which includes both experts and publics.

RRI also calls for a broad range of interdisciplinary expertise in shaping the direction of research and much of the practice of RRI has been focused here, offering opportunities for “trading zones” between different disciplines at the local level of technological development (Murphy, Parry, and Walls 2016). Interdisciplinary collaborations between natural and social scientists can be an opportunity to clarify and develop key questions concerning laboratory animal science and welfare. Working together, social science researchers, animal researchers, and other actors can capture an understanding of “public values” during the innovation process by making differing viewpoints more explicit and feeding back information about the research and innovation processes to broader societal actors. This is clearly a feature of EPSRC-funded synthetic biology centers in the UK, where social science involvement has become integrated into large natural science and engineering projects (see Owen and Goldberg 2010). Kerr (2012) argues that interdisciplinarity presents an important opportunity for “matters of care” to become actionable within RRI and for Science and Technology Studies scholars to work collaboratively with scientists to help prioritize aspects of care within research and innovation. When Russell and Burch (1959) first introduced the 3Rs, they urged social sciences and humanities researchers to play a part in humane experimental design in the animal laboratory (see Kirk 2018). However, interdisciplinary work can be difficult, raising concerns about participation, communication, and the importance of supporting logistics and mediation for the different disciplines (Gunnarsdottir et al. 2012). RRI suggests a potential solution through the embedding of social science and humanities scholars within animal use facilities. There are some examples in other areas of technoscience, where this has been productive in facilitating collaborative and situated critical reflection, allowing a combination of epistemological approaches between scientists and social researchers. This “midstream modulation” approach seeks to build capacity in science and innovation for versatile reflection and responsiveness to a range of societal perspectives throughout the research process (Fisher, Mahajan, and Mitcham 2006; Schuurbiers 2011).

Third, the analysis highlights the neglect of nonhuman animals within RRI. While we argue that RRI can be useful for animal research governance, we also want to draw attention to its anthropocentric focus. The “preface” to *Responsible Innovation* briefly describes how science and innovation might be conducted taking into account: “a greater moral dimension, to those living now, those yet to be born, and *those beyond our own species*” (Owen, Bessant, and Heintz. 2013, xix, emphasis added). Stilgoe, Owen, and Macnaghten (2013) also signpost animal experimentation as an area covered procedurally through existing governance structures. However, fundamental questions about responsibility to nonhuman actors within research and innovation pathways have not been explored thus far, and that is an important area for future research.

## Conclusion

Laboratory animal research governance relies heavily on the 3Rs to demonstrate responsibility. Yet, this interpretation and practice of responsibility is challenged in this highly contested space. Too often, a 3Rs approach to responsibility closes down opportunities to challenge the political dimensions of animal research, particularly its purpose. RRI has the potential to helpfully augment the 3Rs in three key ways: involving a broader range of experts and publics in animal research governance; emphasizing the importance for animal research scientists to take societal, and not just role, responsibilities into account; and acknowledging the political questions animal research raises.
